# A Rare Case of Diabetic Nephropathy and Type III Collagen Glomerulopathy

**DOI:** 10.7759/cureus.44284

**Published:** 2023-08-28

**Authors:** Diana Toro, Rabia Khan, Amir Mohammed

**Affiliations:** 1 Internal Medicine, Augusta University Medical College of Georgia, Augusta, USA; 2 Internal Medicine, WellStar Kennestone Regional Medical Center, Marietta, USA

**Keywords:** type iii collagen, glomerular diseases, collagen deposition, hyperglycemia, renal dysfunction, renal biopsy, nephrotic-range proteinuria, diabetic nephropathy, collagenofibrotic glomerulopathy

## Abstract

Collagenofibrotic glomerulopathy (CG) is a poorly understood kidney disease characterized by extensive deposition of abnormal type III collagen within the glomeruli. We report the case of a 25-year-old man with known type I diabetes mellitus who presented to the emergency department with diaphoresis, nephrotic-range proteinuria, hypertension, and elevated creatinine. Renal biopsy revealed combined collagenofibrotic glomerulopathy and diabetic nephropathy. This case highlights the clinicopathological features of type III collagen glomerulopathy and its possible association with diabetic nephropathy.

## Introduction

Collagenofibrotic glomerulopathy (CG) is a rare non-immune mediated renal disease characterized by abnormal type III collagen deposition in the mesangial matrix and subendothelial space, leading to glomerular injury and dysfunction [1]. It presents with hypertension and proteinuria, often progressing to nephrotic syndrome and renal failure. Type III collagen is a structural protein with elastic properties normally found in the interstitium and blood vessels; but not within the glomerulus [2]. The etiology of CG is unclear, and its distinction as purely a renal or systemic pathology is debated [1-3]. With only 100 documented cases in the literature, diagnosis remains challenging due to its overlapping clinical features with other glomerular diseases [3]. The coexistence of CG with diabetic nephropathy (DN) poses a diagnostic dilemma, as both exhibit nephrotic-range proteinuria. Understanding the epidemiology and prevalence of these concurrent pathologies is crucial for appropriate diagnosis and management. We describe a case of concurrent diabetic nephropathy and type III collagen glomerulopathy, providing valuable insight into the clinical features and management of this challenging renal pathology in practice. 

## Case presentation

A 25-year-old Asian American male with well-controlled type 1 diabetes for over 10 years presented to the emergency room from his primary care office for an abnormally elevated creatinine from baseline. He endorsed increased urinary frequency. He denied flank pain, dysuria, abdominal pain, or hematuria. His past medical history included hypertension, hyperlipidemia, and diabetic nephropathy, which was managed by amlodipine (10 mg once per day), valsartan (80 mg once per day), rosuvastatin (40 mg once per day), insulin glargine (40 units nightly), and insulin lispro (15 units with meals). Upon arrival, his vital signs were stable except his blood pressure was 165/94 mm Hg, while he remained afebrile. On physical examination, he was alert and oriented to person, place, time, and situation. No periorbital or lower limb edema was appreciated. No facial dysmorphic features, abnormal nails, or skeletal abnormalities were identified on clinical examination. His family history was negative for kidney disease. 

Urinalysis was positive for protein, glucose, trace ketones, trace blood, and hyaline casts. Liver function tests were within normal limits. His renal profile showed blood urea nitrogen of 45 mg/dL, serum creatinine of 5.31 mg/dL (serum creatinine was 2.68 mg/dL six months prior to admission), and serum albumin of 2.40 g/dL. The anion gap was ​17. Beta-hydroxybutyrate was normal. He was acidotic with a blood pH of 7.29, bicarbonate 18 mmol/L, and chloride 110 mmol/L. The serum sodium and potassium levels were 141 and 4.2 mmol/L, respectively. A complete blood count revealed a white blood cell count of 15.26×109/L, hemoglobin of 11.0 g/L, and platelet count of 282×109/L. Serum glycosylated hemoglobin level was 6.7%. Twenty-four-hour collection of urine showed a protein content of 15.12 g/day. Abdominal ultrasound showed echogenic, minimally enlarged kidneys (the right and left kidneys measured 10.2 cm and 9.6 cm, respectively). 

He was admitted for further management and evaluation of acute kidney injury on chronic kidney disease secondary to prerenal etiology and underlying diabetic nephropathy. His blood pressure was elevated on presentation and during the first few days of admission which necessitated additional medication for optimum blood pressure control. The blood pressure was later optimally controlled with amlodipine (10 mg once a day), carvedilol (25 mg twice a day), and losartan (100 mg once a day) which was started after improvement in kidney function. Serum creatinine trended down from 5.31 mg/dL to 3.42 mg/dL with hydration. He was initially started on an intravenous bicarbonate drip with improvement in metabolic acidosis and then later transitioned to a sodium bicarbonate tablet. Serum C3 and C4 levels were normal (119 mg/dL and 26 mg/dL, respectively). Screening for antinuclear antibodies and anti-neutrophil cytoplasmic antibodies was negative. 

**Figure 1:**
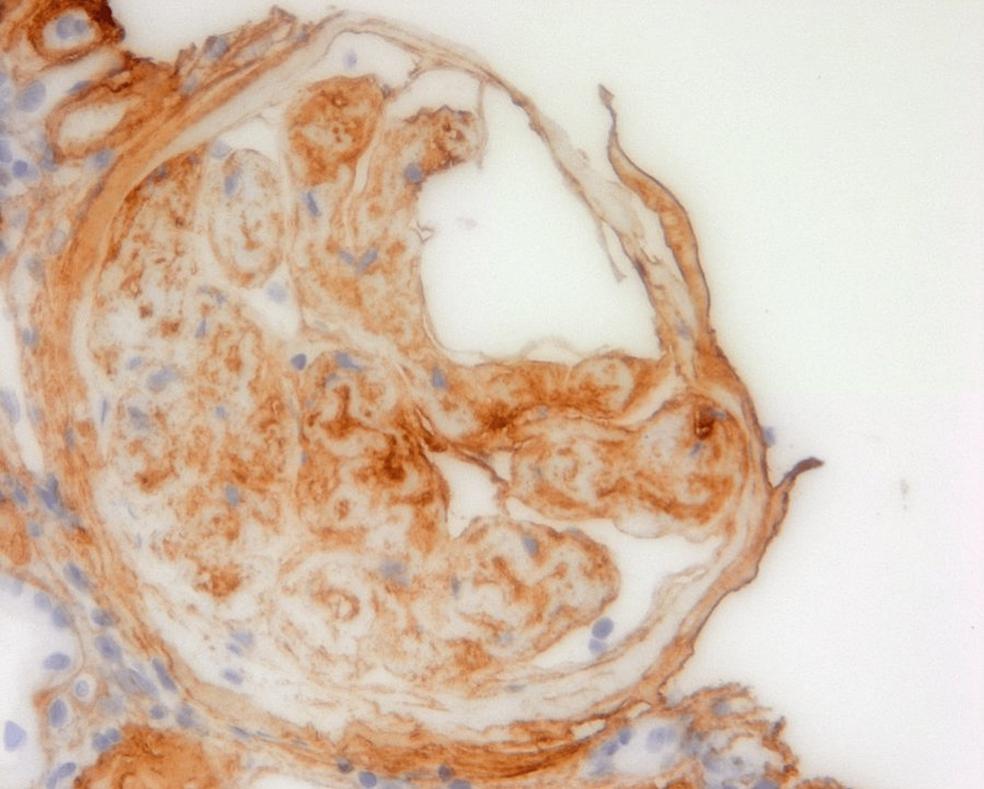
By light microscopy, an immunohistochemical stain for type III collagen shows positive staining in the mesangium and capillary walls.

**Figure 2:**
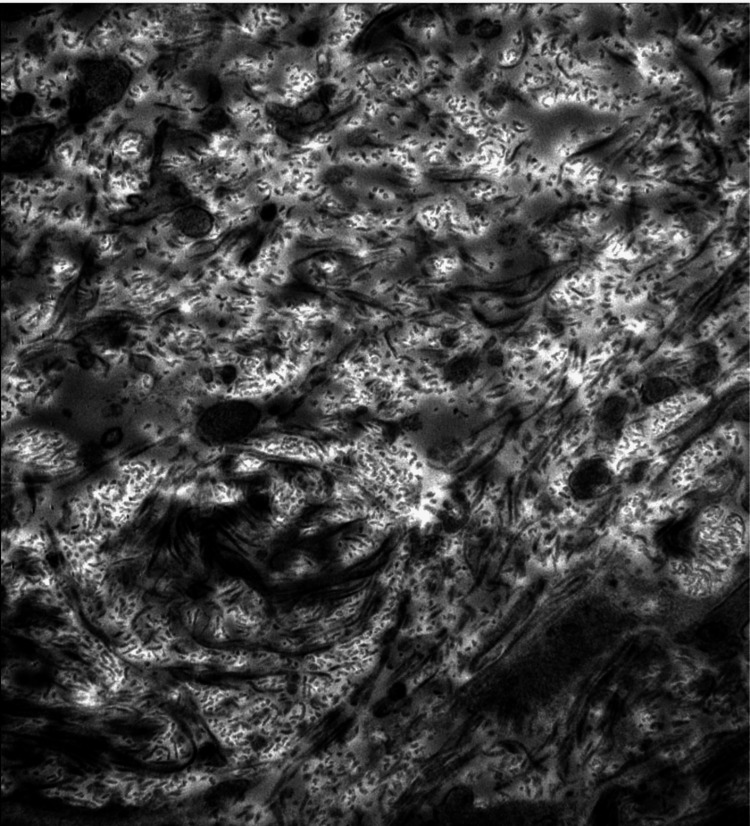
Electron microscopic image showing collagen fibril deposits within the mesangial and subendothelial space.

A CT-guided renal biopsy was performed. Three micrometers thick sections from the kidney tissue were examined under light microscopy, electron microscopy, and immunofluorescence. Light microscopy tissue sections were stained for hematoxylin and eosin, periodic acid Schiff, Jones methenamine silver, silver methenamine with Masson trichrome, and Masson trichrome. Under light microscopy, 41 glomeruli were present, of which 35 were globally sclerosed. Severe interstitial fibrosis and tubular atrophy (IFTA) were present. An immunohistochemical stain for type III collagen showed positive staining in the mesangium and capillary walls (Figure 1). By immunofluorescence microscopy, there were 15 glomeruli examined, of which 13 were globally sclerosed. Among the sclerosed glomeruli, nodular-appearing mesangial expansion was reported by dark-field examination. IgA, IgM, complement C3, kappa, and lambda stained weakly positive. On electron microscopy, amorphous to segmentally fibrillar and curvilinear collagen fibrils, consistent with type III collagen, were identified in the mesangial and subendothelial space. There was marked subendothelial widening and new basement membrane formation. The basement membranes were variably thickened. Severely effaced epithelial foot processes were also noted (Figure 2). After successfully managing his blood pressure and seeing an improvement in kidney function, the patient was eventually discharged.

## Discussion

CG has an intriguing history that traces back to the late 1970s when Arakawa identified the first cases of CG in Japanese patients. These patients exhibited a unique nephrotic syndrome characterized by proteinuria and progressive kidney dysfunction. Histopathological investigations revealed collagen fibrils within the mesangial matrix and subendothelial space [1,3]. CG was initially believed to be a subset of nail-patella (NP) syndrome, a rare genetic disorder characterized by maldeveloped nails, kneecaps, and nephrotic syndrome. However, these early patients did not demonstrate skeletal abnormalities, and NP syndrome was eventually linked to mutations in LMX1B, confirming that CG and NP are similar but distinct entities. In addition, NP syndrome exhibits collagen accumulation in the glomerular basement membrane rather than the mesangium and subendothelial space [4]. Our patient did not exhibit any skeletal abnormalities or endorse a family history of kidney disease. While a distinct genetic inheritance pattern for CG remains undefined, some case reports have indicated a potential genetic influence, particularly when manifesting during childhood rather than adulthood [1,4,5]. In this instance, the disease occurrence seems sporadic; notably, the patient's age is relatively young, at 25 years old. As more cases of CG are identified, gaining a better understanding of its hereditary nature has become imperative. 

Patients with CG typically present with nephrotic-range proteinuria, edema, hypertension, and microscopic hematuria [2]. Its exact epidemiology is not well-established. Existing knowledge about CG comes from sporadic case reports and small case series [1-3]. The onset of CG can occur at any age, but it is often detected in adulthood. Our patient's clinical presentation included nephrotic-range proteinuria, hypertension, trace hematuria, and severe renal dysfunction. CG will progress to renal failure, often within 10 years, but the decline in renal function is more rapid in younger patients [4]. Additionally, this patient has underlying diabetic nephropathy (DN). The patient’s reported symptoms, demographic characteristics, and medical history suggest a potentially severe manifestation of CG. 

The etiology of CG is elusive. The aberrant deposition of type III collagen is thought to be related to a disturbance in collagen synthesis, processing, or degradation [4]. This case report sheds light on the rare occurrence of concurrent DN and CG, emphasizing the importance of recognizing and differentiating these coexisting renal pathologies. The relationship between glucose deposition in DN and collagen deposition in CG is complex and multifactorial. Hyperglycemia in diabetes mellitus triggers the endogenous synthesis of type III collagen by mesangial and glomerular endothelial cells, explaining the presence of glomerulosclerosis in DN. It is unclear if the abnormal synthesis of type III collagen stems from mesangial and endothelial cells or results from an abnormal metabolism of type III collagen on a systemic level [2]. Nevertheless, understanding the relationship between CG and DN can provide insight into the underlying mechanisms and help to devise appropriate management strategies. 

Light microscopy shows that the majority of the glomeruli were globally sclerosed. Under electron microscopy, curvilinear stacked collagen bundles, consistent with type III collagen, were identified in the mesangial and subendothelial space. Severely effaced epithelial foot processes, variably thickened glomerular basement membranes, and increased mesangial matrix with nodule formation were also reported, consistent with diabetic nephropathy. An immunohistochemical stain for type III collagen was also positive within the mesangium, confirming its deposition. 

Currently, no treatment is available for CG. Clinical management focuses on controlling blood pressure, preserving kidney function, and reducing proteinuria. However, end-stage renal disease is inevitable and often occurs within a decade of diagnosis [2,3]. Kidney transplant has successfully cured CG in a few limited cases [4]. 

## Conclusions

Understanding the clinical and pathological features of type III collagen glomerulopathy, along with its challenges in diagnosis and management, is crucial for optimizing patient outcomes. Collaboration among clinicians, researchers, and medical institutions is necessary to gather data on additional cases, contributing to a greater understanding of this rare renal disorder. Further research and case reports will enhance our knowledge of CG's epidemiology, natural history, and long-term outcomes.
